# Supplementary material for: Viral infection and transmission in a large, well-traced outbreak caused by the SARS-CoV-2 Delta variant
Source: Nat Commun. 2022 Jan 24;13:460. doi: 10.1038/s41467-022-28089-y (PMC8786931; doi:10.1038/s41467-022-28089-y)
Supplement: Supplementary file 1 — Supplementary Information [file 41467_2022_28089_MOESM1_ESM.pdf]

Supplementary information for:

Viral infection and transmission in a large, well-traced outbreak caused by the SARS-CoV-2 Delta variant

Baisheng Li[1,2#], Aiping Deng[1,2#], Kuibiao Li[3#], Yao Hu[1,2#], Zhencui Li[1,2#], Yaling Shi[4#], Qianling Xiong[1,2,5], Zhe Liu[1,2,5], Qianfang Guo[1,2], Lirong Zou[1,2], Huan Zhang[1,2],Meng Zhang[1,2], Fangzhu Ouyang[1,2], Juan Su[1,2], Wenzhe Su[3], Jing Xu[1,2], Huifang Lin[1,2,5], Jing Sun[1,2,5], Jinju Peng[1,2,5], Huiming Jiang[1,2,5], Pingping Zhou[1,2,5], Ting Hu[1,2], Min Luo[1,2], Yingtao Zhang[1,2], Huanying Zheng[1,2], Jianpeng Xiao[1,2,5], Tao Liu[1,2,5], Mingkai Tan[4], Rongfei Che[1,2], Hanri Zeng[1,2], Zhonghua Zheng[1,2], Yushi Huang[1,2], Jianxiang Yu[1,2], Lina Yi[1,2,5], Jie Wu[1,2], Jingdiao Chen[1,2], Haojie Zhong[1,2], Xiaoling Deng[1,2], Min Kang[1,2], Oliver G. Pybus[6], Matthew Hall[7], Katrina A. Lythgoe[7], Yan Li[1,2*], Jun Yuan[3*], Jianfeng He[1,2*], Jing Lu[1,2,5*]

[1]Guangdong Provincial Center for Disease Control and Prevention, Guangzhou, Guangdong, China

[2]Guangdong Workstation for Emerging Infectious Disease Control and Prevention, Chinese Academy of Medical Sciences, Guangzhou, Guangdong, China

[3]Guangzhou Center for Disease Control and Prevention, Guangzhou, Guangdong, China

[4]Guangzhou 8th People's Hospital, Guangzhou, Guangdong, China

[5]Guangdong Provincial Institution of Public Health, Guangzhou, Guangdong, China

[6]Department of Zoology, University of Oxford, Oxford OX1 3SZ, UK

[7]Big Data Institute, Nuffield Department of Medicine, University of Oxford, Old Road Campus, Oxford OX3 7LF, UK

[#] These authors contributed equally: Baisheng Li, Aiping Deng, Kuibiao Li, Yao Hu, Zhencui Li, Yaling Shi

[*]Corresponding authors:

Yan Li, 13580581074@126.com

Jun Yuan, yuanjuncom@163.com

Jianfeng He, hjf@vip.sina.com

Jing Lu, Jimlu0331@163.com

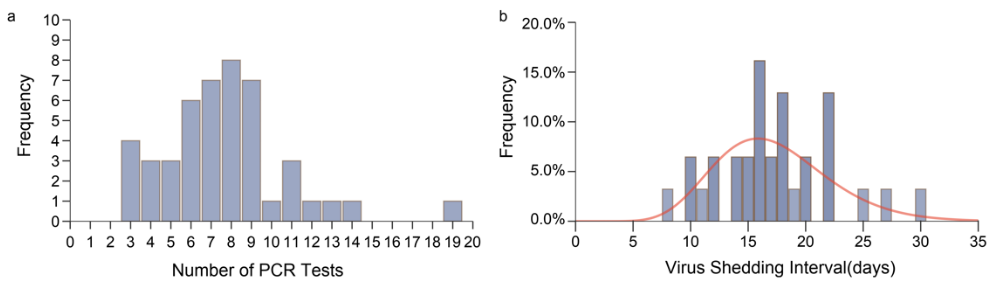

Supplementary Figure 1. Statistical description of epidemiological information. **a** Distribution of frequency of PCR+ test from quarantined individuals. **b** Distribution of virus shedding interval (from first PCR+ test to first PCR- test). Detail testing information was included in supplementary data.

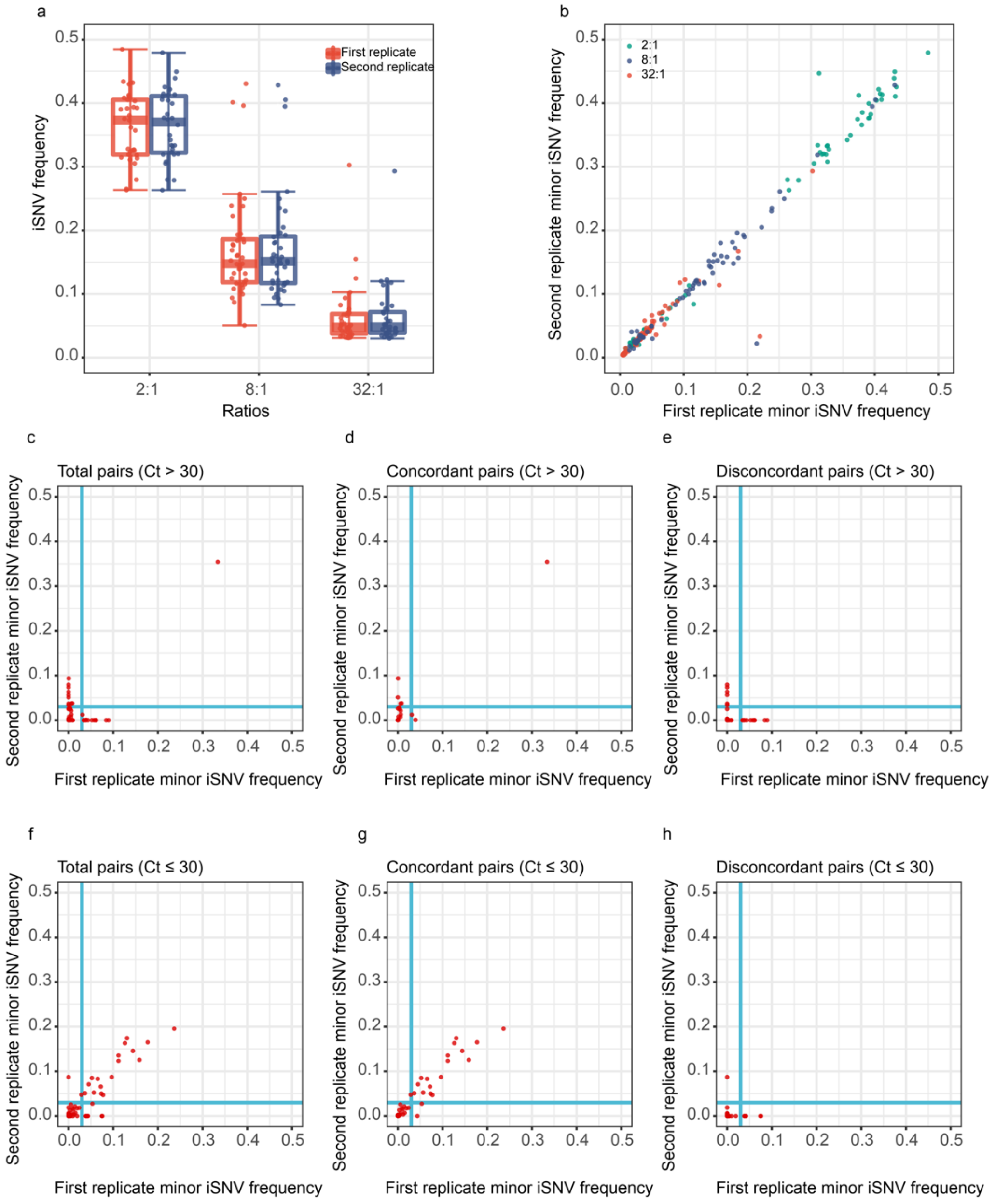

Supplementary Figure 2. The accuracy and reproducibility of calculating iSNVs frequency via multiplex PCR and high throughput sequencing. Two different viral lineage strains (B.1.351 and A) were first quantified by RT-PCR and mixed at different ratios 1:2, 1:8 and 1:32 which Ct (Cycle threshold) value for each group was ~20. We only included the sites which are variant between two inputted strains. **a** The points represent iSNVs frequency identified through multiplex PCR and high throughput sequencing. Box plots indicate the median (middle line), 25th, 75th percentile (box) and 5th and 95th percentile (whisker). The ratio for the minor iSNVs frequency in these variant sites were expected as 0.33, 0.11 and 0.03 according to the viral loads of the input strains. **b** The points represent the iSNVs in each of the replicate pairs. If MAFs are reproducible, we expect a positive correlation. **c-h** Fifteen replicate pairs were performed separate reverse transcription (RT), PCR

amplification, and library preparation steps. Results were shown with sequencing pairs Ct >30 (6 pairs, **c-e**) and Ct ≤30 (9 pairs, **f-h**), respectively. We included sites with alter frequency >=3%, depth >100 and alter depth >10 in at least one of the 30 replicates. The points represent the iSNVs identified in all replicate pairs. If minor iSNVs are reproducible, we expect a positive correlation. The blue line shows 3% iSNVs frequency.

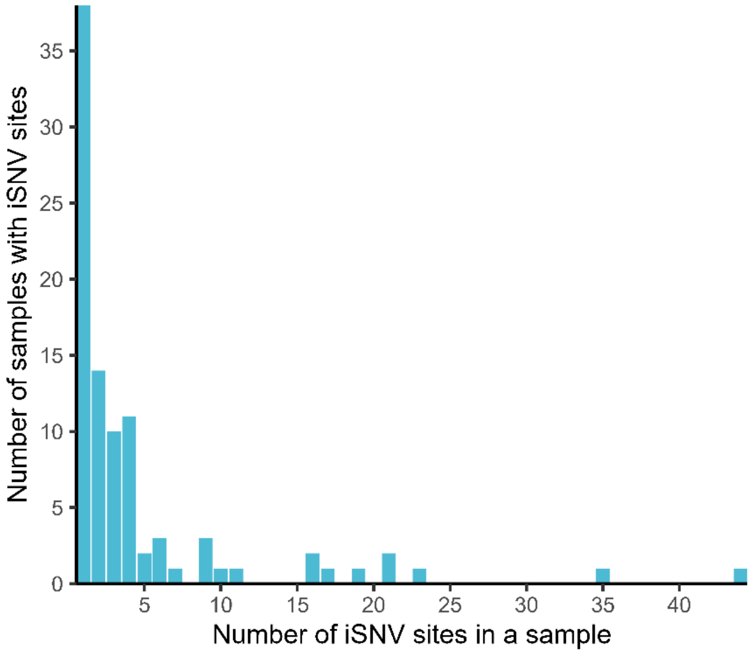

Supplementary Figure 3. Histogram of the number of samples (y-axis) by the number of minor iSNV per sample (x-axis), n = 93.

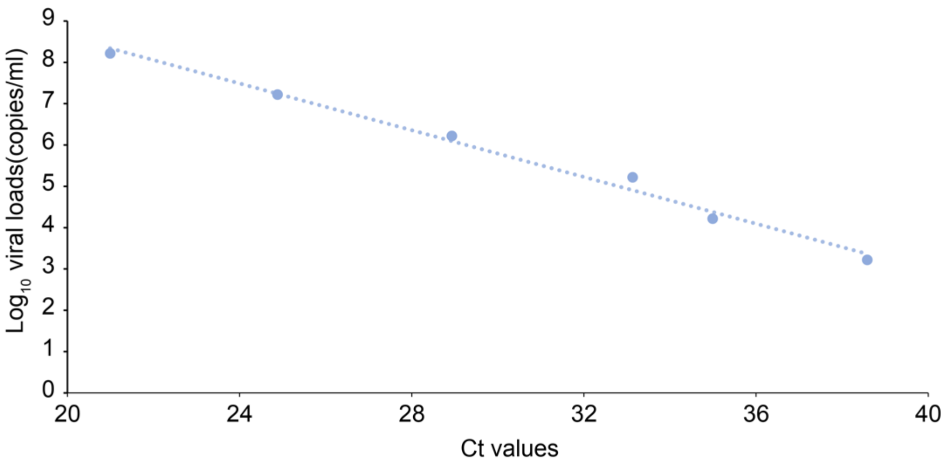

Supplementary Figure 4. Standard curve relationship between virus RNA copies and Ct values. The reference pseudovirus containing gene fragment of SARS-CoV-2 were serially diluted from 1.67×10^7 to 1.67×10^3 copies/mL. The viral RNA was extracted and evaluated in duplicate with RT-qPCR. The best-fit linear regression of the average Ct value on the $\log_{10}$-transformed standard values had slope -0.2829 and intercept 14.279 ($R^2 = 0.99$). The linear regression equation: $y = -0.2829x + 14.279$.

Supplementary Table 1. Ct values of first PCR+ test for cases in 2020 and 2021 epidemic

Case_NO.	CT Value of first PCR+ test	Pangolin_Lineage	Epidemic
SF200	29	B.57	2020
SF117	25	A	2020
SF012	36	A	2020
SF013	35	A	2020
SF5111	38	A	2020
SF1153	36	B	2020
SF273	36	B	2020
SF028	31	B	2020
SF1107	35	B	2020
SF753	31	B	2020
SF123	26	A	2020
SF254	35	A	2020
SF602	40	B	2020
SF7617	35	B	2020
SF629	33	B	2020
SF014	35	B	2020
SF025	36	A	2020
SF315	35	B	2020
SF401	33	B	2020
SF040	30	B	2020
SF1113	34	NA	2020
SF1892	36	NA	2020
SF3004	36	B	2020
SF3006	30	A	2020
SF4207	38	B	2020
SF4320	39	NA	2020
SF4204	36	A	2020
SF4205	38	NA	2020
SF12497	32	B	2020
SF1136	19	B	2020
SF115	33	A	2020
SF665	23	B	2020
SF1152	27	B	2020
SF1159	33	B	2020
SF812	35	B	2020
SF3388	40	B	2020
SF174	36	B	2020
SF190	34	B	2020
SF198	28	B	2020
SF201	34	B	2020

NA: Not determined

Supplementary Table 1. Ct values of first PCR+ test for cases in 2020 and 2021 epidemic

Case_NO.	CT Value of first PCR+ test	Pangolin_Lineage	Epidemic
SF243	40	B	2020
SF5286	40	B	2020
SF1999	24	B	2020
SF4631	35	B	2020
SF2546	33	A	2020
SF316	36	B	2020
SF3646	36	A	2020
SF4319	38	A	2020
SF4047	33	A	2020
SF4051	31	A	2020
SF3646	34	A	2020
SF616	31	B	2020
SF630	38	B	2020
SF5192	33	A	2020
SF5187	32	A	2020
SF632	35	B	2020
SF7782	36	B	2020
SF684	31	A	2020
SF438	24	B	2020
SF2074	35	B	2020
SF1977	32	A	2020
SF5106	34	B	2020
SF5102	23	B	2020
5371	21	B.1.617.2	2021
5571	21	B.1.617.2	2021
5645	17	B.1.617.2	2021
5646	15	B.1.617.2	2021
5648	17	B.1.617.2	2021
5841	18	B.1.617.2	2021
5844	19	B.1.617.2	2021
5976	33	B.1.617.2	2021
5869	25	B.1.617.2	2021
5873	36	B.1.617.2	2021
5860	37	B.1.617.2	2021
5861	21	B.1.617.2	2021
5931	28	B.1.617.2	2021
6047	36	B.1.617.2	2021
6051	27	B.1.617.2	2021
5952	22	B.1.617.2	2021
6050	30	B.1.617.2	2021

NA: Not determined

Supplementary Table 1. Ct values of first PCR+ test for cases in 2020 and 2021 epidemic

Case_NO.	CT Value of first PCR+ test	Pangolin_Lineage	Epidemic
6055	26	B.1.617.2	2021
5984	24	B.1.617.2	2021
5972	13	B.1.617.2	2021
5985	29	B.1.617.2	2021
6056	29	B.1.617.2	2021
6066	20	B.1.617.2	2021
5986	28	B.1.617.2	2021
5992	27	B.1.617.2	2021
5990	31	B.1.617.2	2021
5994	14	B.1.617.2	2021
6176	33	B.1.617.2	2021
6168	29	B.1.617.2	2021
6165	28	B.1.617.2	2021
6172	24	B.1.617.2	2021
6186	31	B.1.617.2	2021
6202	19	B.1.617.2	2021
6201	14	B.1.617.2	2021
6215	17	B.1.617.2	2021
6204	19	B.1.617.2	2021
6203	24	B.1.617.2	2021
6431	16	B.1.617.2	2021
6213	32	B.1.617.2	2021
6212	27	B.1.617.2	2021
6220	19	B.1.617.2	2021
6244	18	B.1.617.2	2021
6443	26	B.1.617.2	2021
6245	26	B.1.617.2	2021
6308	26	B.1.617.2	2021
6306	24	B.1.617.2	2021
6307	29	B.1.617.2	2021
6342	25	B.1.617.2	2021
6341	18	B.1.617.2	2021
6346	25	B.1.617.2	2021
6729	36	B.1.617.2	2021
6464	24	B.1.617.2	2021
8001	31	B.1.617.2	2021
6465	21	B.1.617.2	2021
6735	36	B.1.617.2	2021
8002	36	B.1.617.2	2021
6543	12	B.1.617.2	2021

NA: Not determined

Supplementary Table 1. Ct values of first PCR+ test for cases in 2020 and 2021 epidemic

Case_NO.	CT Value of first PCR+ test	Pangolin_Lineage	Epidemic
6791	18	B.1.617.2	2021
6703	20	B.1.617.2	2021
8003	23	B.1.617.2	2021
6794	21	B.1.617.2	2021
6934	21	B.1.617.2	2021

NA: Not determined

Supplementary Table 2. iSNV pair transmission

Transmission_pairs	Donor	iSNVs (Donor)	Recipient	iSNVs (Recipient)	Transmitted iSNVs*
pair 1	5137	3	5571	2	1
pair 2	5137	3	5371	2	1
pair 3	5137	3	5645	1	1
pair 4	6173	19	6196	3	0
pair 5	5647	1	5841	2	0
pair 6	5647	1	5976	3	0
pair 7	5647	1	6047	0	0
pair 8	5647	1	5869	44	0
pair 9	5646	1	5869	44	0
pair 10	5646	1	5844	0	0
pair 11	5943	21	5945	2	0
pair 12	6056	3	6066	5	0
pair 13	6056	3	6367	0	0
pair 14	5943	21	5984	0	0
pair 15	5943	21	5990	4	0
pair 16	5943	21	6055	1	0
pair 17	5943	21	5986	21	0
pair 18	5943	21	5947	3	0
pair 19	5943	21	5851	0	0
pair 20	5943	21	5859	0	0
pair 21	6056	3	5984	0	0
pair 22	6056	3	5990	4	0
pair 23	6056	3	6055	1	0
pair 24	6056	3	5986	21	0
pair 25	6056	3	5947	3	0
pair 26	6056	3	5851	0	0
pair 27	6056	3	5859	0	0
pair 28	5843	1	6051	0	0
pair 29	5843	1	6190	1	0
pair 30	5843	1	6174	5	0
pair 31	5843	1	6169	7	0
pair 32	5843	1	6175	1	0
pair 33	5843	1	6201	0	0
pair 34	5843	1	6202	3	0
pair 35	5843	1	6205	3	0
pair 36	5843	1	6240	0	0
pair 37	5843	1	6171	0	0
pair 38	5843	1	5989	2	0
pair 39	5843	1	5993	1	0
pair 40	5843	1	6304	4	0

* Donor iSNVs could not be found in recipient.

Supplementary Table 2. iSNV pair transmission

Transmission_pairs	Donor	iSNVs (Donor)	Recipient	iSNVs (Recipient)	Transmitted iSNVs*
pair 41	5843	1	6305	1	0
pair 42	5843	1	6484	2	0
pair 43	5869	44	6200	2	0
pair 44	6174	5	6212	1	0
pair 45	5989	1	6191	3	1
pair 46	5989	1	6300	4	0
pair 47	5993	1	6300	4	0
pair 48	6304	4	6307	2	0
pair 49	6304	4	6306	3	0
pair 50	6305	1	6307	2	0
pair 51	6305	1	6306	3	0
pair 52	6484	2	6666	0	0
pair 53	6217	1	6220	2	0
pair 54	6244	1	6483	1	0
pair 55	6245	1	6465	3	0
pair 56	6244	1	6547	4	0
pair 57	6241	3	6547	4	0
pair 58	6245	1	6547	4	0
pair 59	6300	4	6292	1	0
pair 60	6300	4	6338	3	0

* Donor iSNVs could not be found in recipient.
