# Supplementary material for: Viral infection and transmission in a large, well-traced outbreak caused by the SARS-CoV-2 Delta variant
Source: Nat Commun. 2022 Jan 24;13:460. doi: 10.1038/s41467-022-28089-y (PMC8786931; doi:10.1038/s41467-022-28089-y)
Supplement: Supplementary file 3 — Description of Additional Supplementary Files [file 41467_2022_28089_MOESM3_ESM.pdf]

Description of Additional Supplementary Files

File Name: Supplementary Data 1
Description: Longitudinal PCR testing were performed on 813 oropharyngeal swabs from 46 individuals, ranging from the first PCR+ till the clearance of viruses.
