# Supplementary material for: Viral infection and transmission in a large, well-traced outbreak caused by the SARS-CoV-2 Delta variant
Source: Nat Commun. 2022 Jan 24;13:460. doi: 10.1038/s41467-022-28089-y (PMC8786931; doi:10.1038/s41467-022-28089-y)
Supplement: Supplementary file 5 — Reporting Summary [file 41467_2022_28089_MOESM5_ESM.pdf]

nature portfolio

Corresponding author(s): Jing Lu

Last updated by author(s): Nov 25, 2021

# Reporting Summary

Nature Portfolio wishes to improve the reproducibility of the work that we publish. This form provides structure for consistency and transparency in reporting. For further information on Nature Portfolio policies, see our Editorial Policies and the Editorial Policy Checklist.

## Statistics

For all statistical analyses, confirm that the following items are present in the figure legend, table legend, main text, or Methods section.

| n/a | Confirmed | |
|---|---|---|
| ☐ | ☒ | The exact sample size (*n*) for each experimental group/condition, given as a discrete number and unit of measurement |
| ☐ | ☒ | A statement on whether measurements were taken from distinct samples or whether the same sample was measured repeatedly |
| ☐ | ☒ | The statistical test(s) used AND whether they are one- or two-sided<br>*Only common tests should be described solely by name; describe more complex techniques in the Methods section.* |
| ☒ | ☐ | A description of all covariates tested |
| ☐ | ☒ | A description of any assumptions or corrections, such as tests of normality and adjustment for multiple comparisons |
| ☐ | ☒ | A full description of the statistical parameters including central tendency (e.g. means) or other basic estimates (e.g. regression coefficient) AND variation (e.g. standard deviation) or associated estimates of uncertainty (e.g. confidence intervals) |
| ☐ | ☒ | For null hypothesis testing, the test statistic (e.g. *F*, *t*, *r*) with confidence intervals, effect sizes, degrees of freedom and *P* value noted<br>*Give P values as exact values whenever suitable.* |
| ☒ | ☐ | For Bayesian analysis, information on the choice of priors and Markov chain Monte Carlo settings |
| ☒ | ☐ | For hierarchical and complex designs, identification of the appropriate level for tests and full reporting of outcomes |
| ☒ | ☐ | Estimates of effect sizes (e.g. Cohen's *d*, Pearson's *r*), indicating how they were calculated |

*Our web collection on statistics for biologists contains articles on many of the points above.*

## Software and code

Policy information about availability of computer code

| | |
|---|---|
| Data collection | No software was used for field data collection. The high-throughput sequencing data were generated by BGI MGISEQ-2000 and Illumina Miniseq. |
| Data analysis | Pipeline for sequencing data from BGI platform (https://github.com/MGI-tech-bioinformatics/SARS-CoV-2_Multi-PCR_v1.0);<br>Illumina data: fastp 0.20.1, BWA 0.7.17 , iVar 1.3.1; the pipeline was deposit in https://github.com/Jinglu1982/Delta-variant-outbreak-in-GZ;<br>Phylogeney construction and visualization: phyml 3.0, nextstrain pipeline for SARS-CoV-2 (https://github.com/nextstrain/ncov),ggtree 2.4.1<br>Pipeline for transmission bottleneck analysis: https://github.com/koellelab/betabinomial_bottleneck |

For manuscripts utilizing custom algorithms or software that are central to the research but not yet described in published literature, software must be made available to editors and reviewers. We strongly encourage code deposition in a community repository (e.g. GitHub). See the Nature Portfolio guidelines for submitting code & software for further information.

## Data

Policy information about availability of data

All manuscripts must include a data availability statement. This statement should provide the following information, where applicable:

- Accession codes, unique identifiers, or web links for publicly available datasets
- A description of any restrictions on data availability
- For clinical datasets or third party data, please ensure that the statement adheres to our policy

All sequencing reads mapped to the reference sequence (the sequences of the first index case, XG5137_GZ_2021/5/21) have been deposited to the GSA database of National Genomics Data Center (https://bigd.big.ac.cn/) with submission number CRA004896 (https://ngdc.cncb.ac.cn/gsa/browse/CRA004896). The generated consensus sequences were submitted with accession number GWHBDIM01000000 – GWHBDNH01000000 (https://ngdc.cncb.ac.cn/gwh/jbrowse/index), and also

shared to NCBI GeneBank with accession number OL663920 – OL664045 (https://www.ncbi.nlm.nih.gov/nuccore). The data underlying Supplementary Figure 1 is provided in Supplementary data 1.

# Field-specific reporting

Please select the one below that is the best fit for your research. If you are not sure, read the appropriate sections before making your selection.

☒ Life sciences ☐ Behavioural & social sciences ☐ Ecological, evolutionary & environmental sciences

For a reference copy of the document with all sections, see nature.com/documents/nr-reporting-summary-flat.pdf

# Life sciences study design

All studies must disclose on these points even when the disclosure is negative.

| | |
|---|---|
| Sample size | Sample sizes were not predetermined prior to the study. There was a total of 167 local infections identified in this outbreak. For sequencing, we used all obtained samples and 126 of 167 high-quality genome (>95% genome coverage) sequences were used in sequence analysis. We investigated all quarantined individuals and 62 individuals detected as positive through the daily sequential PCR testing. Forty six individuals have longitudinal PCR testings based on the hospital surveillance data and included to estimate the viral shedding interval and the viral RNA trajectory of the Delta infections. |
| Data exclusions | Intra-family transmission pairs were removed from our time interval analysis if the exact exposure time for the intra-family transmissions was difficult to pinpoint. |
| Replication | All clinical samples were detected and sequenced in real-time meaning that sample sequencing, as most of other genetic surveillance studies, could not be performed in replicate or triplicate during the outbreak. To test the accuracy of the sequencing method, we did duplicate sequencing on 20 patient samples of which 15 generated a relative high-quality genome sequences (>95% genome coverage) and were included into analysis. This criteria (>95% genome coverage) were pre-established and followed in both phylogenetic and iSNVs analyses. |
| Randomization | N/A as we performed an observational study to generate hypotheses on quarantined cases to investigated the viral load trajectory and no experimental groups were defined in this study. Thus, randomization was not applicable. |
| Blinding | N/A as no group allocation was performed. This was an observational study to generate hypotheses and study individuals were not allocated to experimental groups, thus binding to group allocation was not necessary. |

# Reporting for specific materials, systems and methods

We require information from authors about some types of materials, experimental systems and methods used in many studies. Here, indicate whether each material, system or method listed is relevant to your study. If you are not sure if a list item applies to your research, read the appropriate section before selecting a response.

## Materials & experimental systems

| n/a | Involved in the study | |
|---|---|---|
| ☒ | ☐ | Antibodies |
| ☐ | ☒ | Eukaryotic cell lines |
| ☒ | ☐ | Palaeontology and archaeology |
| ☒ | ☐ | Animals and other organisms |
| ☐ | ☒ | Human research participants |
| ☒ | ☐ | Clinical data |
| ☒ | ☐ | Dual use research of concern |

## Methods

| n/a | Involved in the study | |
|---|---|---|
| ☒ | ☐ | ChIP-seq |
| ☒ | ☐ | Flow cytometry |
| ☒ | ☐ | MRI-based neuroimaging |

## Eukaryotic cell lines

Policy information about cell lines

| | |
|---|---|
| Cell line source(s) | Vero E6 cells (CRL-1586, ATCC) |
| Authentication | The cell line was not authenticated. |
| Mycoplasma contamination | all cells were tested negative for mycoplasma before being used for viral isolation |
| Commonly misidentified lines (See ICLAC register) | None of the cell lines used in this study have been identified as commonly misidentified lines. |

# Human research participants

Policy information about studies involving human research participants

| | |
|---|---|
| Population characteristics | Overall, 46 individuals were performed longitudinal PCR testing with 22 individuals form males and 24 individuals form females. The median age of 46 individuals is 44 years, ranging from 1 years to 75 years. |
| Recruitment | Once a case was detected as SARS-CoV-2 RNA positive based on population screen or hospital surveillance, the individual was reported as an index case and isolated. The close contacts of the index cases were traced and centrally isolated. All quarantined individuals were investigated and have daily PCR testing during the 14 days after exposure. Sixty two infections were identified in the quarantined individuals and included into analyses. |
| Ethics oversight | This study was approved by the institutional ethics committee of the Guangdong Provincial Center for Disease Control and Prevention (GDCDC). Written consent was obtained from patients or their guardian(s) when samples were collected. Patients were informed about the surveillance before providing written consent, and data directly related to disease control were collected and anonymized for analysis. |

Note that full information on the approval of the study protocol must also be provided in the manuscript.
